# Qualitative analysis of biosurfactants from *Bacillus* species exhibiting antifungal activity

**DOI:** 10.1371/journal.pone.0198107

**Published:** 2018-06-04

**Authors:** Ambrin Sarwar, Günter Brader, Erika Corretto, Gajendar Aleti, Muhammad Abaidullah, Angela Sessitsch, Fauzia Yusuf Hafeez

**Affiliations:** 1 Department of Biosciences, Faculty of Sciences, COMSATS Institute of Information Technology, Islamabad, Pakistan; 2 Health & Environment Department, Bioresources Unit, AIT Austrian Institute of Technology GmbH, AIT, Konrad Lorenz Strasse, Tulln, Austria; Dong-A University, REPUBLIC OF KOREA

## Abstract

*Bacillus* spp. produce a broad spectrum of lipopeptide biosurfactants, among which surfactin, iturin and fengycin are widely studied families. The goals of this study were to characterize the biosurfactant activity of *Bacillus* spp. and to investigate their motility and biofilm formation capabilities. In addition, we extracted lipopeptides from these bacteria to assess their antifungal activities and analyzed these products by mass spectrometry (MS). *B*. *amyloliquefaciens* FZB42, *Bacillus* sp. NH 217 and *B*. *subtilis* NH-100 exhibited excellent biosurfactant and surface spreading activities, whereas *B*. *atrophaeus* 176s and *Paenibacillus polymyxa* C1225 showed moderate activity, and *B*. *subtilis* 168 showed no activity. Strains FZB42, NH-100, NH-217, 176s and CC125 exhibited excellent biofilm formation capabilities. Lipopeptide extracts displayed good antifungal activity against various phytopathogens and their associated diseases, such as *Fusarium moniliforme* (rice bakanae disease), *Fusarium oxysporum* (root rot), *Fusarium solani* (root rot) and *Trichoderma atroviride* (ear rot and root rot). Lipopeptide extracts of these strains also showed hemolytic activity, demonstrating their strong potential to produce surfactants. LCMS-ESI analyses identified the presence of surfactin, iturin and fengycin in the extracts of *Bacillus* strains. Thus, the strains assayed in this study show potential as biocontrol agents against various *Fusarium* and *Trichoderma* species.

## Introduction

*Bacillus* species produce a wide variety of antifungal and antimicrobial compounds [1], making them ideal biological control agents [2]. The ability of different *Bacillus* species to prevent several plant diseases has resulted in the commercial development and registration of several *Bacillus* pest management bioproducts that can be integrated into pest management regimens to efficiently control plant diseases [3]. *B*. *amyloliquefaciens*, *B*. *subtilis*, and *B*. *atrophaeus* have the potential to produce secondary metabolites, particularly cyclic lipopeptides, such as iturin, fengycin and surfactin, which have tremendous potential applications in the agriculture, pharmaceutical and biotechnology industries due to their dynamic surface properties [4, 5].

Cyclic lipopeptides (CLPs) are an important class of biosurfactants that have surface-active properties and antifungal activities [6, 7]. These biosurfactants may also have diverse roles in the growth and survival of *Bacillus* strains by increasing the surface area and bioavailability of hydrophobic substrates. In addition, they are involved in biofilm formation, pathogenesis and motility [8, 9]. CLPs are synthesized by non-ribosomal peptide synthetases (NRPs), which generate significant variation among the CLP products with respect to their amino acid sequence and length, branched chain fatty acids and types of peptide bonds [10]. Surfactins, one of the three families of CLP families, are heptapeptides that interlink to form a cyclic lactone ring structure with a β-hydroxy fatty acid and have a role in motility and biofilm formation. The iturin family includes seven isomers, including mycosubtilin and bacillomycin, which are heptapeptides linked from C_14_ to C_17_ to a β-amino fatty acid chain [10]. Iturins have strong hemolytic and antifungal activities [11]. The third CLP family contains fengycins A and B, which are lipodecapeptides with a β-hydroxy fatty acid chain from C_14_ to C_18_ with antifungal activity that can be unsaturated or saturated [10].

Mass spectrometric analyses are used to identify secondary metabolites in different microbes [12], as it is an efficient and reliable approach [13]. Differences in various *Bacillus* spp. have been observed with respect to CLP production [14]. CLPs are frequently effective against *Collectotrichum dematium* [15], *Rhizoctonia solani* [16], *Pythium ultimum* [17] and *Fusarium graminearum* [18].

Our group previously isolated phytopathogen antagonists from the rhizosphere of sugarcane and identified *B*. *subtilis* NH-100 and *Bacillus* sp. NH-217 as surfactin producers [19, 20]. In the current study, *B*. *amyloliquefaciens* FZB42, a previously reported lipopeptide-producing strain [21] was used reference strain, while *B*. *subtilis* 168 was used as a negative control. The goals of this study were: (1) to evaluate the biosurfactant activities of *Bacillus* spp.; (2) to assess the presence/absence of lipopeptides produced by the assayed *Bacillus* spp.; and (3) to assess the production and antifungal activities of lipopeptides produced by *Bacillus* spp. at specific culturing time intervals.

## Materials and methods

### Bacterial and fungal strains

Bacterial strains capable of producing lipopeptides, *B*. *subtilis* NH-100 (EU627167) and *Bacillus* sp. NH-217 (EU627170) [19], and the fungal strain *F*. *moniliforme* KJ719445 were obtained from the Applied Microbiology and Biotechnology (AM&B) Lab at the COMSATS Institute of Information Technology (CIIT), in Islamabad, Pakistan. The lipopeptide-producing strain *B*. *amyloliquefaciens* FZB42 (NC009725) and *B*. *subtilis* 168, which is unable to produce surfactin, were used as reference strains and were obtained from the BGSC (*Bacillus* Genetic Stock Center). *B*. *atrophaeus* 176s [22] and *Paenibacillus polymyxa* CCI25 [23] were provided by the Health and Environment Department, Bioresources unit, AIT Austrian Institute of Technology GmbH. Bacteria were stored at -80°C in 20% glycerol and streaked on Luria-Bertani (LB) agar plates to obtain isogenic colonies [24]. After incubating overnight at 28°C, colonies of *Bacillus* strains were collected from LB agar plates with disposable loops and used to initiate 10 mL LB broth starter cultures.

*Bacillus* strains were morphologically characterized by their growth on Lauria Bertani (LB) agar plates (Somasegaran and Hoban, 1994) and LB broth. Purification of *Bacillus* colonies was confirmed by Gram’s staining, motility and phenotypic characteristics.

The fungal strain *F*. *oxysporum* was obtained from Dr. Attique (Punjab University), while *F*. *solani* SAN1077 and *T*. *atroviride* P150907 were provided by the Austrian Institute of Technology (AIT). Fungal cultures were streaked onto PDA plates with a disposable loop and incubated at 28°C for 5–8 days, after which a single fungal colony was subcultured onto a fresh PDA plates for purification.

### *Bacillu*s motility, aggregation and biofilm formation

*Bacillus* species were evaluated for swimming and swarming activity at 28°C by spotting 2.5 μL of bacterial cell suspensions (1 x 10^8^ CFU mL^-1^) onto LB medium (Miller, Fluka) supplemented with 0.3% or 0.5% agar technical (Oxoid), respectively. In each analysis, motility was recorded as the diameter (cm) of *Bacillus* spp. displacement from the inoculation point after 24 and 48 h.

The ability of *Bacillus* strains to form aggregates may significantly affect their persistence in the plant rhizosphere, dispersal and adsorption to roots. Aggregation was evaluated as described by Madi and Henis [25] with slight modifications and with six replicates for each strain. Aliquots of cultures were transferred to test tubes containing aggregant, followed by a 30 min incubation at room temperature. The OD of aggregates at the bottom of each test tube and the culture turbidity was recorded with a Synergy 5 microplate reader (BioTek Instrument Inc.; Winooski, USA) at 540 nm. *Bacillus* cultures were disrupted for 1 min with a tissue homogenizer and the ODtt (total turbidity) was recorded. The percent aggregation was assessed as follows:

%aggregation=(ODtt−ODts)×100÷ODtt

Where

ODtt = OD of total turbidity; and

ODts = OD of turbidity of each suspension.

Biofilm formation was evaluated using *Bacillus* cultures that were grown overnight in 96-well microtiter plates. To quantify the amount of biofilm formation, cells were stained with crystal violet (1%) for 45 min and then were washed with 200 μL of absolute ethanol. The absorbance of the stained cells was then recorded at 595 nm [26].

### Biosurfactant activity of *Bacillus* strains

#### Hemolytic activity

Hemolytic activity was assessed by inoculating 2 μL of overnight *Bacillus* cultures onto blood agar plates (BBL™ Trypticase™ Soy Agar (TSA II) with 5% Sheep Blood) and incubated at 28°C for 72 h. During the 12–72 h incubation, the formation of halo zones was observed on blood agar plates. *Bacillus* strains with clear halo zones were recorded as biosurfactant-producing strains [27].

#### Bacterial adherence to hydrocarbons (BATH) activity

The adherence of *Bacillus* strains to hydrocarbons was assessed by measuring the cell hydrophobicity according to the method described by Rosenberg et al. [28]. Cell pellets were collected from *Bacillus* cultures grown in LB medium for 72 h. The pellets were washed twice with a solution consisting of K_2_HPO_4_ (16.9 g L^-1^) and KH_2_PO_4_ (7.3 g L^-1^), then were resuspended in the same buffer solution and diluted to an OD of 0.5 at 600 nm. Crude oil (100 μL) was added to the cell suspension (4 mL) in test tubes and vortexed for 3 min. After shaking, the aqueous phase and the crude oil were allowed to stand for 2 h. Next, the OD_610_ of the aqueous phase was measured with a Nano Drop instrument (ND-1000, USA). The percentage of *Bacillus* cells adhered to the crude oil was determined from the OD values according to the formula:

%Bacillusadherence={1−(ODhydrocarbons÷ODcontrol)}×10

Where

OD hydrocarbons = the OD of the *Bacillus* cells and the crude oil mixture; and

OD control = the OD of the bacterial cell suspension before mixing with crude oil in the buffer solution.

#### Oil spreading activity

Oil spreading activity was determined using the method described by Morikawa et al. [29] by adding 25 μL of crude oil to a petri plate containing 25 mL of distilled water. *Bacillus* cultures were grown in LB medium for 72 h at 28°C, after which the cultures were centrifuged and 10 μL of cell-free bacterial supernatant was added to the surface of the oil. If the oil layer was evacuated with a clearing zone having no oil, the cell-free supernatant exhibited biosurfactant activity. Distilled water was used as negative control in the experiment.

#### Emulsification activity

Bacterial strains were grown in LB broth at 28 ± 2°C for 72 h, after which the culture broth was incubated for 20 min at 121°C and centrifuged for 20 min at 5000 rpm to obtain cell-free culture broth (crude biosurfactants) for the biosurfactant activity assays. One milliliter of cell-free culture broth and 10 mg of hydrocarbon were added to 5 mL of 50 mM Tris buffer (pH 8.0) in a test tube and vortexed for 2 min. The emulsion was incubated at room temperature for 40 minutes and the OD was recorded at 610 nm using a spectrophotometer [28]. The negative control consisted of the buffer solution mixed with crude oil, and Triton X-100 was used as a positive control.

#### Drop collapse assay

The drop collapse assay was performed by the Bodour and Miller-Maier method [30]. Crude oil was added to the wells of 96-well microplate and was allowed to equilibrate for 24 h. Cell-free culture broth (5 μL) was added to the wells coated with oil and the drop size was recorded after one minute with the aid of a magnifying glass. Diameters larger than 1 mm were recorded as showing biosurfactant activity, with 1 mg/mL of Triton X-100 used as a control.

### Growth curves, hemolytic and antagonistic activity of *Bacillus* strains

*Bacillus* strains (*B*. *amyloliquefaciens* FZB42, *B*. *subtilis* NH-100, *Bacillus* sp. NH-217, *B*. *atrophaeus* 176s and *Paenibacillus polymyxa* CCI25) were grown at 28 ± 2°C in an LB or Landy medium. Overnight bacterial cultures (500 μL) were inoculated into 250-mL flasks containing 50 mL of LB or Landy medium. Landy medium consists mineral salts and nutrients (Glucose 20 g/L, L-glutamic acid 5 g/L, MgSO4 0.5 g/L, KCl 0.5 g/L, KH2PO4 1.0 g/ L, Fe2SO3 0.0012 g/L, MnSO4 0.0014 g/L, CuSO4 0.0016 g/L) which enhance lipopeptide production. Therefore, LB and Landy medium was selected for comparision of lipopeptides activities. The flasks were incubated on a rotary shaker at 28°C and 160 rpm. One milliliter aliquots of the cultures were withdrawn at different times (24, 48 and 72 h) and were used to determine: the optical density (OD_600nm_), hemolytic activity as described in section 2.3.1; and the antifungal activity of the lipopeptides present in the cell-free supernatants. Lipopeptides were extracted by acid precipitation with concentrated HCl and were suspended in 800 μL of Milli-Q^®^ water for 30 min. To assess the antagonistic activity, the extracted lipopeptides were suspended in 80% (v/v) ethanol and 200 μL of each extract was spread onto PDA plates. The antagonistic activity of the crude lipopeptides was tested against different phytopathogenic fungi using the percentage inhibition method [19]. Fungus that grew in the absence of any inhibitory substance was used as control. For each fungus, a fungal plug was positioned in the middle of the PDA plates. After an incubation for 7 days at 28 ± 2°C, the diameter of mycelium growth was measured. Fungal growth inhibition was assessed by comparing the percent reduction of mycelium growth with respect to control PDA plates, with experiments executed in triplicate. The percent inhibition was calculated according to the formula described by Sivan et al. [31]:

PercentageofInhibition={1−(GrowthofFungus×ControlGrowth)}×100

### Surface tension of lipopeptides produced by *Bacillus* species

The surface tension of *Bacillus* cultures grown in LB medium was measured at 24, 48 and 72 h of growth. Lipopeptides were extracted by acid precipitation with concentrated HCl and suspended for 40 min in 800 μL of Milli-Q^®^ water. The surface tension was measured by the Wilhelmy method with a MicroTroughX instrument (Kibron Inc.; Espoo, Finland) [32]. The surface tension of distilled water (72 mN m^−1^) was taken as a reference.

### Qualitative analysis of extracted lipopeptides

Lipopeptide production of *Bacillus* cultures grown for 72 h was determined by centrifuging cultures at 4000 rpm for 15 min. Lipopeptides were precipitated from culture supernatants by adding concentrated HCl to a pH 2.0 and were refrigerated overnight at 4°C. The precipitates were collected by centrifugation at 4000 rpm for 15 min followed by lyophilization at -50°C for 24 h. The lyophilized powder was extracted with 4 mL methanol for 4 h. The methanol extract was then filtered through 0.45-μm filters and stored at -20°C until being analyzed. Qualitative analyses were performed by LCMS-ESI. The lipopeptide extract was directly injected into the Mass Spectrometer (LTQ XL Linear Ion Trap Mass Spectrometer) using a direct insertion pump equipped with an ESI source (Thermo Scientific, USA). Samples were run with a flow rate of 10 μL min^-l^ in both positive and negative mode. Sheath gas flow (N_2_) and capillary temperature were set in both scan modes at 350°C and 30 arbitrary units. The results were achieved in both negative and positive total ion full scan mode with mass scan range of *m*/*z* 50–2000.

### Statistical analysis

Analysis of variance (ANOVA) of the experimental data was calculated using the Statistix software (version 8.1 for Windows, Chicago, UK). Mean values of the data were compared and separated on the basis of Fisher’s least significant difference (LSD) test.

## Results

Morphological identification revealed that the colony size of *Bacillus* strains was large to moderate, colony colour was white, cream, brownish and greyish white whereas margins were irregular exept in *Paenibacillus polymyxa* (CC125) in which margins were entire circular (S1 Table). All bacterial strains were gram positive, rod and circular (CC125) shaped and their optimum growth temperature was 30–37°C. Endospore formation was present in all strains and colony elevation was flat, raised and convex (S1 Table).

### Motility, biofilm formation and aggregate stability of lipopeptide-producing *Bacillus* strains

To assess the bacterial motility of the selected *Bacillus* strains, swarming and swimming tests were performed on 0.7 and 0.3% agar-solidified LB plates, respectively, according to Bindel Connelly et al. [33]. The strains FZB42, NH-100 and NH-217 showed further spreading from the inoculation sites compared to the 176s, CC125 and 168 strains. FZB42 exhibited highest aggregation ability (40%), whereas NH-100 showed an aggregation of 36%. Strains NH-217, 176s, and CC125 showed 33, 24, 13% aggregation. No aggregate formation was observed for strain 168. Biofilm formation was highest in the FZB42 strain, while strains NH-100, NH-217,176s and CCI25 also exhibited good biofilm formation (Table 1).

**Table 1:** Motility and biofilm formation of the lipopeptide-producing *Bacillus* species at different time intervals.

LPB strains	Surface spreading ability (cm)				Biofilm		% Aggregation
	Swimming activity		Swarming activity		OD 600 nm	OD 595 nm	
	24 h	48 h	24 h	48 h			
***B*. *amyloliquefaciens* FZB42**	1.16^a^± 0.057	1.73^a^± 0.057	3.33^c^± 0.152	5.36^c^± 0.152	0.95^a^ ± 0.002	0.77^a^± 0.006	40.13^a^± 1.35
***B*. *subtilis* NH-100**	1.03^b^ ± 0.115	1.2^b^ ± 0.1	4.50^a^ ± 0.2	6.16^a^ ± 0.115	0.69^e^ ± 0.007	0.68 ^b^± 0.007	35.81^b^± 0.630
***Bacillus* sp. NH-217**	1.23^a^ ± 0.057	1.83^a^ ± 0.057	3.76^b^ ± 0.115	5.70^b^ ± 0.173	0.78^c^ ± 0.002	0.53^c^± 0.004	33.14^c^± 0.909
***B*. *atrophaeus* 176s**	0.66 ^c^ ± 0.057	0.66^c^ ± 0.057	1.033^d^ ± 0.115	1.033^d^ ± 0.115	0.82^b^ ± 0.002	0.30^e^ ± 0.001	24.02^d^ ± 0.011
***Paenibacillus polymyxa* CCI25**	0.33^e^ ± 0.057	0.33^e^ ± 0.057	1.033^d^ ± 0.115	1.033^d^ ± 0.115	0.42^f^ ± 0.016	0.32^d^ ± 0.007	13.13^e^ ± 0.017
***B*. *subtilis* 168**	0.466^d^ ± 0.057	0.466^d^ ± 0.057	0.467^e^ ± 0.057	0.467^e^ ± 0.057	0.66^d^ ± 0.003	0.256^f^ ± 0.001	11.08^f^ ± 0.011

Presented values are the means ± standard deviations and different letters from a-f in the same columns indicate a significant difference from each other according to the analysis of variance (p < 0.05).

Experiments were repeated in triplicate with three replicates.

### Biosurfactant activity of *Bacillus* species

Hemolytic activity

Out of the six *Bacillus* strains assayed, five tested positive for hemolytic activity. *B*. *amyloliquefaciens* FZB42 (reference strain) and *B*. *atrophaeus* 176s showed the maximum hemolytic activity (1.8 cm halo zone), while *Paenibacillus* CCI25 showed the least activity (0.5 cm) (Table 2). *B*. *subtilis* strains NH-100 and NH-217 exhibited moderate hemolytic activity (1 cm), whereas no activity was observed for *B*. *subtilis* 168.

**Table 2:** Biosurfactant properties of lipopeptide-producing *Bacillus* species.

LPB strains	Hemolytic assay (cm)	BATH assay (%)	Oil Spreading assay (cm)	Emulsification assay (OD 610nm)	Drop Collapse assay
***B*. *amyliliquefaciens* FZB42**	1.8^a^ ± 0.057	89^a^ ± 0.577	2.7^a^ ± 0.057	2.7^a^ ± 0.057	+++
***B*. *subtilis* NH-100**	1^a^ ± 0.057	90^a^ ± 0.577	2.9^a^ ± 0.461	2.9^a^ ± 0.057	+++
***Bacillus* sp.NH-217**	1^a^ ± 0.057	88^a^ ± 0.577	1.6^b^ ± 0.057	2.8^a^ ± 0.057	++
***B*. *atrophaeus* 176s**	1.8^a^ ± 0.057	79^b^ ± 0.577	0	2.4^a^ ± 0.057	-
***Paenibacillus polymyxa* CC125**	0.3^b^ ± 0.057	77^b^ ± 0.577	0	0	-
***B*. *subtilis* 168**	0	0	0	0	-

Values represent the means ± standard deviations and different letters a-b in the same columns are significantly different from each other according to the analysis of variance (p < 0.05).

‘+++’ Excellent, ‘++’ Good, ‘+ ‘Fair ‘-’ Absent. All experiments were repeated in triplicate with three replicates.

#### Drop collapse assay

The drop collapse method involves assaying a small volume (~5 μL) of culture broth for biosurfactant activity. Among the six strains tested, three strains (FZB42, NH-100 and NH-217) tested positive (Table 2). The maximum drop collapse activity was observed for strains FZB42 and NH-100 (drop collapse within 1 min), whereas for strain NH-217 the drop collapse occurred after 1 min. Strains 176s, 168 and CC125 were negative for drop collapse activity.

#### Oil spreading activity

The results of the oil spreading test were in agreement with the results of the drop collapse assay (Table 2). Strains positive for drop collapse (FZB42, NH-100 and NH-217) also tested positive for oil spreading activity. These results confirmed the presence/absence of biosurfactants in the cell free supernatants.

#### Emulsification activity

The results of the emulsification assay revealed that four out of six strains tested positive for emulsification activity. The emulsification activity for strains FZB42, NH-100, NH-217 and 176s were 2.7, 2.9, 2.8 and 2.4 at OD_610_, respectively. No emulsification activity was observed for strainsCC125 and 168 (Table 2).

#### BATH activity

The ability of cells to adhere to crude oil (hydrocarbon mixture) for the tested bacterial strains ranged from 77 to 90%. The maximum cell attachment was observed for the strains NH-100 (90%), FZB42 (89%), NH-217 (88%), and 176s (2.4%), whereas no activity was observed for strains CC125 and 168 (Table 2).

### Growth curves, hemolytic activity and fungal growth inhibition by lipopeptide-producing *Bacillus* (LPB) species

*Bacillus* strains showed almost identical growth curves, reaching stationary phase in approximately 24 h. Their final optical densities were also similar, approximately an OD of 0.7 (A_620_) in both LB and Landy media. For hemolytic and antifungal assays, mid-exponential growth was assayed at 11 h, early stationary phase growth was assayed at 24 h and late stationary phase growth was assayed at 48 h.

Lipopeptide-producing *Bacillus* (LPB) strains exhibited remarkable hemolytic activity after 48 and 72 h when grown alone and co-cultured with different fungal strains (*F*. *moniliforme*, *F*. *oxysporum*, *F*. *solani* and *Trichoderma atroviride*) in LB and Landy medium. Maximum halo zones with a diameter of 1.8 cm were observed in treatments with FZB42, NH-100, and 176s. Interestingly, hemolytic activity in for the NH-100 strain was normal the in absence of fungal supernatant, but increased (1.8 cm) in co-culture treatments. *Paenibacillus polymyxa* also showed good hemolytic activity (1 cm halo zone) with LB and Landy media in co-culture treatments. No hemolytic activity was observed for *B*. *subtilis* 168 (Table 3).

**Table 3:** Hemolytic activity upon co-inoculation of LPB with pathogenic *Fusarium* and *Trichoderma* species.

*LPB strains	**24 h**	**48 h**	**72 h**	**96 h**
LB FZB42	─	++	+++	+++
LB NH-100	─	++	+	+
LB NH-217	─	+	++	++
LB168	─	─	─	─
LB 176s	─	+	+	+
LB CCI25	─	─	─	─
Landy FZB42	─	+	+++	+++
Landy NH-100	─	++	+++	+++
Landy NH-217	─	+	++	++
Landy 168	─	─	─	─
Landy 176s	─	+++	+++	+++
Landy CCI25	─	+	+	+
**LPB+Fungal supernatant**				
LB FZB42	─	++	++	++
LB NH-100	─	+++	+++	+++
LB NH-217	─	+	++	++
LB168	─	─	─	─
LB 176s	─	+++	+++	+++
LB CCI25	─	─	─	─
Landy FZB42	─	─	++	++
Landy NH-100	─	+++	+++	+++
Landy NH-217	─	+	++	++
Landy 168	─	─	─	─
Landy 176s	─	+++	+++	+++
Landy CCI25	─	++	++	++

*All LPB strains were grown in Landy and LB broth.

Samples were taken at four time intervals for hemolytic assays, where: ‘+++’ indicates a halo zone of 1.1–1.8 cm, ‘++’ indicates a halo zone of 0.5–1 cm, ‘+’ indicates a halo zone of 0.1–0.3 cm, and ‘NA’ indicates no halo zone was observed. Experiments were repeated in triplicate with three replicates.

Lipopeptide producing *Bacillus* (LPB) species exhibited significant antifungal activity against pathogenic fungal strains of *F*. *moniliforme*, *F*. *oxysporum*, *F*. *solani* and *Trichoderma atroviride*. The different levels of activity against various phytopathogenic fungi are as shown in Table 4. *B*. *amyloliquefaciens* FZB42 showed the maximum inhibition, up to 83% in LB medium and 87% in Landy medium. *B*. *subtilis* NH-100 inhibited up to 79% in LB medium and 80% in Landy medium. NH-217 also showed good antagonistic activity, inhibiting the growth of these fungi by up to 76% in LB medium and 79% in Landy medium. *B*. *atrophaeus* 176s showed inhibition of up to 67% in LB medium and 62% in Landy medium. *Paenibacillus* CC125 also exhibited antagonistic activity against *F*. *solani* and *Trichoderma*, up to 57 and 55%, respectively. The lowest fungal inhibition (3–5%) was observed for strain 168 (Table 4).

**Table 4:** Antifungal activity of crude lipopeptide extract of *Bacillus* species on different growth media.

% Fungal growth inhibition								
LPB strains	LB medium				Landy medium			
	1	2	3	4	1	2	3	4
**FZB42**	77^a^±0.57	83^a^±0.57	79^a^±0.57	75^b^±0.61	80^a^±0.57	85^a^±0.57	87^a^±0.57	77^a^±0.57
**NH-100**	71^b^±0.62	79^b^±0.57	73^b^±0.0.61	79^a^±0.17	73^c^±0.57	80^b^±0.57	75^c^±0.57	71^b^±0.57
**NH-217**	66^c^±0.57	76^c^±0.57	68^c^±0.15	67^c^±0.57	77^b^±0.57	79^c^±0.57	78^b^±0.57	68^c^±0.57
**176 s**	56^d^±0.57	67^d^±0.05	56^d^± 1.15	55^d^±0.57	61^d^±0.57	61^d^±0.57	61^d^±0.57	62^d^±0.57
**CC125**	54^e^±0.57	56^e^±0.57	53^e^±0.57	52^e^±0.57	58^e^±0.57	57^e^±0.57	56^e^±0.57	55^e^±0.57
**168**	03^f^±0.57	04^f^±0.57	04^f^±0.57	05^f^±0.57	04^f^±0.57	05^f^±0.57	05^f^±0.57	04^f^±0.57

Values represent the means ± standard deviations and different letters a-f in the same columns are significantly different from each other according to the analysis of variance (p < 0.05).

1: *F*. *moniliforme*, 2: *F*. *oxysporum*, 3: *F*. *solani*, 4: *T*. *atroviride*. Experiments were repeated in triplicate with three replicates.

### Surface tension of lipopeptide-producing *Bacillus* species

**Fig 1:**
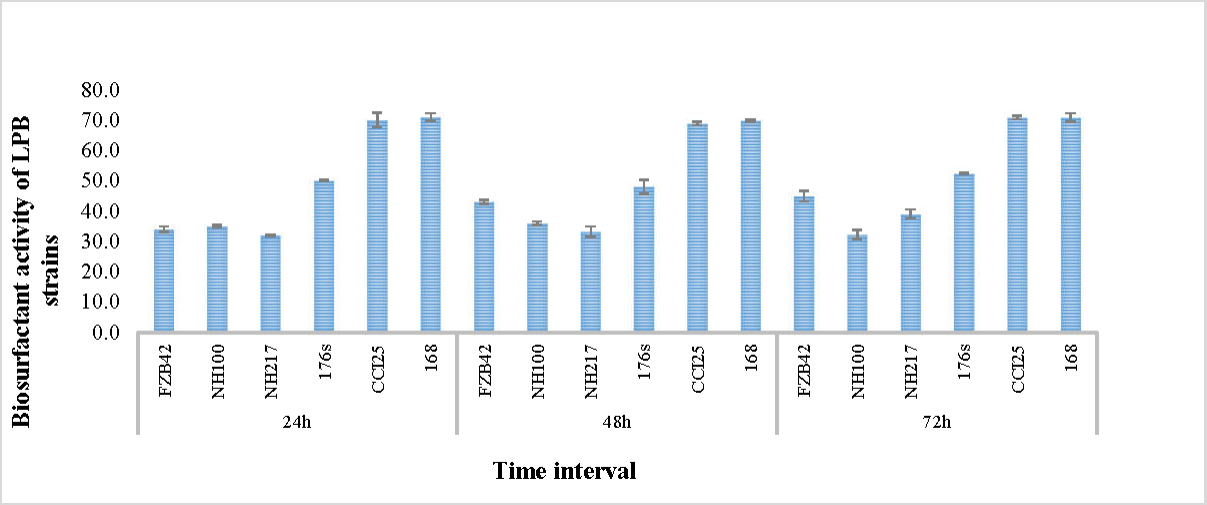
Biosurfactant activity of lipopeptide-producing *Bacillus* species at different time intervals. Bars indicate the standard error from three replicates. All treatments are significantly different from each other according to the analysis of variance (p < 0.05). Experiments were repeated in triplicate with three replicates. *Bacillus* strains were grown in the optimum medium for lipopeptide production at 28°C. The surface tension of the water was 72 mN m^-1^.

To assess the biosurfactant activity of lipopeptides in the supernatants of the *Bacillus* species, lipopeptide and water mixtures were made to measure surface tension. According to the data shown in Fig 1, after a 24 h incubation the surface tension of water was reduced from 72 mN m^-1^ to approximately 43.23 mN m^-1^ for strain FZB42, 36.10 mN m^-1^ for strain NH-100, 33.40 mN m^-1^ for strain NH-217, 48.11 mN m^-1^ for strain 176s, 69 mN m^-1^ for strain CC125 and 70 mN m^-1^ for strain 168. Lipopeptides produced after 72 h of culturing decreased the surface tension of water to approximately 45 mN m^-1^ by strain FZB42, 32.40 mN m^-1^ by strain NH-100, 39.25 mN m^-1^ by strain NH-217, 52.67 mN m^-1^ by strain 176s and 71 mN m^-1^ by strains CC125 and 168 (Fig 1).

### Mass spectrometric analysis of lipopeptide-producing *Bacillus* species

**Fig 2:**
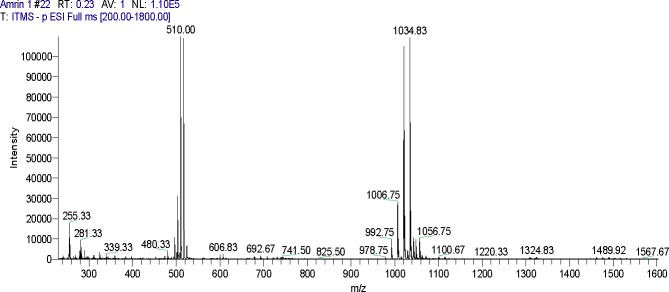
MS-ESI scan of lipopeptides produced by *B*.*subtilis* NH-100.

**Fig 3:**
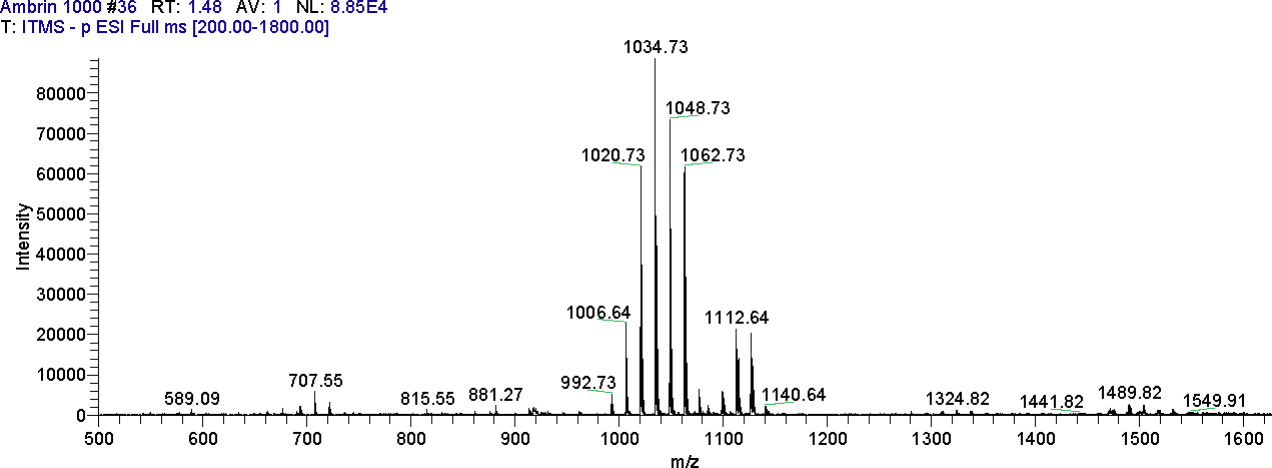
MS-ESI scan of lipopeptides produced by *B*. *atrophaeus* 176s.

Filtrates of *B*. *subtilis* NH-100 and *B*. *atrophaeus* 176s, which exhibited the highest antifungal and hemolytic activities, were assayed by LCMS-ESI, which showed intense peaks of surfactin, fengycins and low abundant peaks of iturin (Figs 2 and 3). The ESI-MS analysis revealed that the first set of strong signal peaks belonged to the surfactin family at *m/z* 1008, 1030.8, 1044.8 and 1058.6 and fragile [M^+^H] ^+^ signals of these surfactin variants occurred at *m/z* 994.8, 1008.8, 1022.8 and 1050.6. These peaks indicated the presence of isomers with C_12_, C_13_, C_14_, C_15_ and C_16_ acyl chains, respectively.

The iturin peaks showed four primary [M^-^H]−signals at *m/z* 1042.6, 1056.6, 1072.7 and 1083.8. The molecular weights of these molecules correspond to the molecular weights of iturin A isomers with C_14_, C_15_, C_16_ and C_17_ acyl chains, respectively (Table 5). The third set of peaks belonged to the fengycin family, with molecular weights of these molecules corresponding to the molecular weights of fengycin A and B with C_14_, C_15_, C_16_ and C_18_ acyl chains.

**Table 5:** Cyclic lipopeptides produced by *Bacillus* species (NH-100, NH-217 and 176s) detected by ESI-MS.

Lipopeptides	Exact mass m/z	Observed peaks			Isomers
		[M^+^H]^+^	[M^+^Na]^+^	[M-H]-	
**Surfactin**	93	994	1008	992	C12
	1007	1008	1030	1006	C13
	1021	1022	1044	1020	C14
	1035	1036	1058	1034	C15
	1049	1050	1072	1048	C16
	1063	-	1086	1062	C17
**Iturin**	1043	-	1066	1042	C14
	1057	-	1080	1056	C15
	1073	1074	1096	1072	C16
	1084	-	1106	1083	C17
**Fengycin**	1435	1436	-	1434	C14
	1450	1451	-	1449	C15
	1492	1490	-	1491	C16
	1521	-	-	1521	C18

Cyclic lipopeptides were identified by comparing both actual masses and detected peaks with those from the literature (Ongena et al. 2005; Ongena and Jacques 2008).

## Discussion

Lipopeptides have been studied at an accelerated rate in the last decade due to their antimicrobial, antifungal, immunosuppressant, antitumor and biosurfactant activities [34]. Lipopeptides of bacterial origin are ideal due to several traits, such as their low or non-toxicity, environmental friendliness, high surface activity and specificity, activity under extreme conditions and are easy to recycle compared to synthetic surfactants [35, 36]. Subsequently, they are broadly used, including in the cosmetic, food, agriculture and pharmaceutical industries [36]. Lipopeptides help bacteria to survive and grow by allowing them to establish of multicellular communities, supporting increased colonization, motility and swarming abilities [37]. The rapid production of lipopeptides can also destabilize swarming activity of bacterial strains through genetic control of the *swr* operon [38]. Luo [39] reported that surfactin and bacillomycin L impart synergistic effects on the phenotypic topographies of *B*. *subtilis* 916 and rice sheath blight biocontrol caused by *Rhizoctonia solani*. Production of the lipopeptides surfactin and mycosubtilin was directly correlated with improved spreading on a swarming medium [39]. These lipopeptides enhance surface spreading and decrease surface tension. Moreover, they are beneficial during the surface colonization of plants. The improved spreading of the lipopeptide-producing *Bacillus* strains on semi-solid agar medium observed in this study was possibly due to increased lipopeptide production (Table 1), especially surfactin, which could enable faster colonization in a dual culture assay.

Colonization and survival of bacteria in the environment of plants is essential for the promotion of plant growth and yield. Motility, biofilm formation and aggregate stability are significant characteristics that promote the colonization of root surfaces [40]. It has been suggested that a criterion for the active colonization of roots by bacteria is they provide a favorable environment for bacterial motility [41]. Strains FZB42, NH-100 and NH-217 tested positive for motility, which likely plays an important role in root colonization. Biofilm formation and aggregation were common characteristics in the all strains tested in this study (FZB42, NH-100, NH-217, 176s, CC125 and 168). Three strains (FZB42, NH-100 and NH-217) tested positive for swimming and swarming motility (Table 1).

The hemolytic assay is commonly used to characterize the production of biosurfactants and it is the only technique used to characterize biosurfactant production [42, 43]. Bernheimer and Avigad [44] reported that red blood cells were lysed due to biosurfactants, such as surfactin, produced by *B*. *subtilis*. A direct relationship between biosurfactant production and hemolytic activity was observed by Carrillo et al. [45]. Previous studies [46, 47] have indicated the possibility of biosurfactant production without associated hemolytic activity. However, the presence or absence of hemolytic activity may be due to strong or weak biosurfactant producers [48]. It is noteworthy that not all biosurfactants possess hemolytic activity, and some additional compounds having no biosurfactant activity may cause hemolysis. Thus, in the present study the BATH assay, drop collapse test, oil spreading assay and emulsification assay were performed to detect the biosurfactant activity of *Bacillus* strains.

Positive hemolytic, drop collapse and BATH assay results showed that strains FZB42, NH-100, NH-217, 176s and CC125 could produce biosurfactants and have a high cell hydrophobicity (Table 2). Biosurfactant-producing strains (FZB42, NH-100, NH-217 and 176s) tested positive in the drop collapse and BATH assays, whereas the *B*. *subtilis* 168 did not exhibit biosurfactant activity. These results suggest that it is important to evaluate the biosurfactant activity of both the whole cells and the corresponding cell free culture broths. The precision and consistency of the drop collapse assay observed in the present investigation are in agreement with the findings of Bodour and Miller-Maier [30].

The oil displacement area in the oil spreading assay is directly proportional to the biosurfactant concentration in the solution [29]. While exploring microbes for biosurfactant production, similar results were observed by Youssef et al. [46]. In the emulsification assay, it was presumed that if the biosurfactants were present in cell-free culture broth, then hydrocarbons present in the testing solution will be emulsified. The results of this assay showed that four out of six strains (FZB42, NH-100, NH-217 and 176s) exhibited emulsification activity. Thus, hemolytic, BATH and drop collapse assays could be used to verify the production of biosurfactants (Table 2). The use of these assays could be an ideal approach to replace expensive LCMS-ESI analyses.

The results of biosurfactant activity assays of *Bacillus* supernatants showed that the growth time interval having the highest biosurfactant activity did not correlate with the highest antifungal activity (Fig 1, Tables 2 and 4 and S2 Table). This result suggests that the change in the lipopeptide composition during growth may result in a higher antifungal activity and a subsequent decrease in biosurfactant activity, or possibly that some other biosurfactant compounds are being released to the medium before 72 h of culture that have no antifungal activity (Tables 2 and 4 and S2 Table) [49].

Lipopeptides extracted from cell-free culture broth of strains FZB42, NH-100, NH-217, 176s and CC125 displayed a broad-range of antifungal activities. Lipopeptide extracts significantly suppressed the growth of the tested fungal pathogens, especially *F*. *moniliforme*, *F*. *solani*, *F*. *oxysporum* and *Trichoderma atroviride* (Table 4). In addition to *Bacillus*, some isolates of the closely related *Paenibacillus* genus also exhibited effective antifungal activity against different phytopathogens, such as *F*. *moniliforme*, *F*. *oxysporum*, *F*. *solani* and *T*. *atroviride* (Table 4). We therefore compared the hemolytic and antifungal activity of *Paenibacillus polymyxa* CC125 with the other *Bacillus* strains. The antifungal activity assay showed that CC125 exhibits a good spectrum of antagonistic activity against fungal pathogens compared to that of the most efficient *Bacillus* strains assayed (Table 4).

Filtrates of *B*. *subtilis* NH-100, *Bacillus* sp. NH-217 and *B*. *atrophaeus* 176s, which had the highest antifungal and hemolytic activities, were analyzed by LCMS-ESI (Fig 2 and Fig 3). LC-HRMS (/MS) was performed by Aleti et al. [22] on filtrates of strain 176s, which showed intense peaks of surfactin and fengycins and low abundant peaks of iturin. In the present study, the filtrates of strain 176s were subjected to an LCMS-ESI analysis, along with those strain NH-100 for a comparison of lipopeptide families.

According to our results, the first set of observed peaks corresponded to compounds belonging to the surfactin family and represented homologues containing C_12_, C_13_, C_14_, C_15_ and C_16_ acyl chains (Table 5 and Fig 2). Apparent iturin peaks were observed at negative and positive ionization modes, which supports the novelty of the current study and the method of choice. The molecular weights of these fragments were in accordance with those of isomers of iturin A containing C_14_, C_15_, C_16_ and C_17_ acyl chains, whereas the molecular weights observed for fengycin are also in accordance with fengycin A containing C_14_, C_15_, C_16_ and C_18_ acyl chains (Table 5). These LCMS-ESI results are in agreement with previous studies by Ali et al. [50] and Aleti et al. [22]. Previous studies reported that some *B*. *amyloliquefaciens* and *B*. *subtilis* strains could co-produce fengycin, iturin and surfactin [51, 52]. However, to investigate the individual roles of surfactin, iturin and fengycin, an efficient purification method is required because the co-production of lipopeptides makes purification difficult. As different lipopeptides are produced by different *Bacillus* strains, their biocontrol potential varies against different phytopathogens [53]. Simple and efficient purification methods emphasize the potential application of these lipopeptide-producing *Bacillus* (LPB) strains as a biocontrol agents.

The results of the present study demonstrates the potential of biosurfactants produced by *Bacillus* spp. that produce surfactins, iturins and fengycins. The study describes a novel, simple and easy to perform approach to screen surfactant-producing bacteria that does not require more expensive techniques, such as LCMS-ESI. Moreover, the novel lipopeptides exhibited tremendous antifungal activity against *Fusarium* and *Trichoderma* species. Effective co-production of the three lipopeptide families (surfactins, iturins and fengycins) is thus clearly beneficial and should be considered as basic criteria to select potential lipopeptide-producing *Bacillus s*trains as biocontrol agents against different phytopathogens.

## Supporting information

S1 Table Morphological characterization of *Bacillus* strains. (DOCX)

S2 Table Biosurfactant production at different time intervals in LB and Landy media. (DOCX)

S1 Fig AJE certificate. (PDF)
