# Correction: Qualitative analysis of biosurfactants from *Bacillus* species exhibiting antifungal activity

**DOI:** 10.1371/journal.pone.0201624

**Published:** 2018-07-26

**Authors:** Ambrin Sarwar, Günter Brader, Erika Corretto, Gajender Aleti, Muhammad Abaid Ullah, Angela Sessitsch, Fauzia Yusuf Hafeez

The fourth and fifth authors’ names are spelled incorrectly. The correct names are: Gajender Aleti and Muhammad Abaid Ullah. The correct citation is Sarwar A, Brader G, Corretto E, Aleti G, Ullah MA, Sessitsch A, et al. (2018) Qualitative analysis of biosurfactants from *Bacillus* species exhibiting antifungal activity. PLoS ONE 13(6): e0198107. https://doi.org/10.1371/journal.pone.0198107
