# Supplementary material for: Qualitative analysis of biosurfactants from Bacillus species exhibiting antifungal activity
Source: PLoS One. 2018 Jun 4;13(6):e0198107. doi: 10.1371/journal.pone.0198107 (PMC5986119; doi:10.1371/journal.pone.0198107)
Supplement: S1 Table — (DOCX) [file pone.0198107.s001.docx]

**S1 Table Morphological characterization of *Bacillus* strains**

| **Characteristics** | ***B. amyloliquefaciens***  **FZB42** | ***B. subtilis***  **NH-100** | ***B. subtilis***  **NH-217** | ***B. subtilis* 168** | ***B. atrophaeus* 176s** | ***Paenibacillus* CCI-25** |
| --- | --- | --- | --- | --- | --- | --- |
| **Colony Size** | large | large | large | moderate | large | moderate |
| **Colony Colour** | white | cream | white | brownish | brownish | greyish-white |
| **Surface** | mucoid | mucoid | mucoid | mucoid | mucoid | mucoid |
| **Margin** | irregular | irregular | irregular | irregular | irregular | entire circular |
| **Bacterial Cell Shape** | rod | rod | rod | rod | rod | circular |
| **Endospore Formation** | present | present | present | present | present | present |
| **Elevation** | raised | flat | raised | flat | flat | convex |
| **Growth in Liquid Medium(LB broth)** | turbid | pellicle | pellicle | sediment | pellicle | sediment |
| **Motility** | + | + | + | - | - | - |
| **Growth Optimum Temperature** | 37 | 37 | 37 | 37 | 37 | 30 |
| **Growth pH** | 7 | 7 | 7 | 7 | 7 | 7 |
