# Supplementary material for: Qualitative analysis of biosurfactants from Bacillus species exhibiting antifungal activity
Source: PLoS One. 2018 Jun 4;13(6):e0198107. doi: 10.1371/journal.pone.0198107 (PMC5986119; doi:10.1371/journal.pone.0198107)
Supplement: S2 Table — (DOCX) [file pone.0198107.s002.docx]

**S2 Table Biosurfactant production at different time intervals in LB and Landy media**

| **LPB strains** | **Time** | **LB medium (mg/L)** | **Landy medium (mg/L)** |
| --- | --- | --- | --- |
| **FZB42** | 24h | 85 ± 14.1 | 592 ± 31.2 |
|  | 48h | 192 ± 11.3 | 1025 ± 65.6 |
|  | 72h | 216 ± 13.2 | 1140 ± 49.1 |
| **NH-100** | 24h | 34 ± 6.2 | 861 ± 13.2 |
|  | 48h | 103 ± 14.8 | 1603 ± 32.4 |
|  | 72h | 196 ± 4.1 | 1830 ± 45.4 |
| **NH217** | 24h | 45 ± 54.2 | 757 ± 52.1 |
|  | 48h | 167 ± 62.7 | 1011 ± 9.0 |
|  | 72h | 183 ± 64.9 | 1104 ± 45.2 |
| **176s** | 24h | 60 ± 102.1 | 448 ± 53.8 |
|  | 48h | 756 ± 51.2 | 936 ± 62.9 |
|  | 72h | 982 ± 45.4 | 1011 ± 40.1 |
| **CCI25** | 24h | 21 ± 11.3 | 93 ± 14.7 |
|  | 48h | 49 ± 9.2 | 117 ± 13.9 |
|  | 72h | 53 ± 5.7 | 121± 5.0 |
| **168** | 24h | 01 ± 0.577 | 01 ± 0.577 |
|  | 48h | 02 ± 0.577 | 02 ± 0.577 |
|  | 72h | 02 ± 0.577 | 01 ± 0.577 |

Values represent the means ± standard deviations and experiments were repeated in triplicate with three replicates.

.
